# Prospective Study: Utility of Anterior Segment Optical Coherence Tomography to Identify Predictive Factors of Recurrence in Pterygium Surgery

**DOI:** 10.3390/jcm13164769

**Published:** 2024-08-14

**Authors:** Marina Aguilar-González, Enrique España-Gregori, Isabel Pascual-Camps, Luis Gómez-Lechón-Quirós, Cristina Peris-Martínez

**Affiliations:** 1 Unit of Cornea and Anterior Eye Diseases, Fundación de Oftalmología Médica (FOM), C/Pío Baroja 12, 46015 Valencia, Spain; cristinaperismartinez0@gmail.com; 2 Hospital de Manises, Av. De la Generalitat Valenciana 50, 46949 Manises, Spain; 3 Hospital Universitario y Politécnico La Fe, Av. Fernando Abril Martorell 106, 46026 Valensia, Spain; enrique.espana@uv.es (E.E.-G.); i.pascualcamps@gmail.com (I.P.-C.); 4 Department of Surgery, Ophthalmology, Universitat de Valencia, Avenida Blasco Ibáñez 15, 46010 Valencia, Spain; 5 Hospital Francesc de Borja, Gandía, 46702 Valencia, Spain; lgomezlechon@gmail.com; 6 Aviñó Peris Eye Clinic, Avenida del Oeste 34, 46001 Valencia, Spain

**Keywords:** pterygium, recurrence, ocular surface imaging, anterior segment ocular coherence tomography, AS-OCT

## Abstract

**Background/Objectives:** The main purpose of this study is to determine, by anterior segment optical coherence tomography (AS-OCT), the anatomical characteristics, both preoperatively and postoperatively, that correlate with a higher rate of pterygium recurrence after surgery with exeresis and conjunctival autograft with biological glue. **Methods:** A total of 50 eyes which were listed for primary pterygium surgery at an ophthalmology tertiary centre were treated with standard pterygium excision and a conjunctival autograft with tissue glue. Ten variables were measured with AS-OCT (Casia 2; Tomey Corp., Nagoya, Japan) during six control visits with all patients. Finally, statistical analysis was performed using SPSS (SPSS stadistics^®^, IBM^®^, version 21.0.0.0) for descriptive variables and R-project (The R foundation©, version 3.0.2) for the rest of the analyses, including a descriptive analysis and an inferential analysis studying prognostic factors of recurrence and their predictive capacity. **Results:** Among the 50 patients who underwent surgery, recurrence was detected in 8 cases (rate 16%; 95% CI: 5.8–26.2%). Most cases (n = 6) were detected 3 months after surgery. The pattern of recurrences was atrophic in two thirds of the cases; none required reintervention. Preoperative total conjunctival thickness at 3 mm was significantly increased in patients who developed recurrence. One week after surgery, epithelial and stromal thickness at 1 mm and total thickness at 3 mm proved to be useful for predicting recurrence. Both models have significant discriminant capacity. **Conclusions:** By imaging the graft with AS-OCT preoperatively and 7 days after surgery, the risk of future recurrence can be predicted.

## 1. Introduction

Pterygium is a non-neoplastic, fibrovascular growth of the ocular surface [1]. It is frequently found in patients from Central American countries, where a prevalence of up to 22% has been reported in the general population. In countries of northern and southern latitudes, prevalence rates approach 2% of the general population, and the lesion mainly affects patients with increased exposure to sunlight, usually related to work activity [2,3,4]. Surgical removal of pterygium is indicated in cases of visual impairment caused by irregular astigmatism, chronic ocular irritation, restricted eye movements, or cosmetic disfigurement [5,6,7]. However, despite the possibility of excision, postoperative recurrence is frequent, as it can occur in up to 50% of cases, depending on the treatment used, and often leads to more aggressive clinical behaviour [8].

Anterior segment optical coherence tomography (AS-OCT) is a relatively new imaging method that allows real-time, artifact-free quantitative imaging of biological tissue structures with high resolution, and it is useful in the diagnosis and management of many ocular surface pathologies [9], including pterygium. A high concordance has been demonstrated between AS-OCT images and histopathological findings of pterygium [10], and AS-OCT allows measurements of pterygium to be obtained more accurately and with more repeatability than by slit lamp examination (SLE) [7]. As a result, tomographic features of pterygium have been described in several studies. However, few predictive factors of pterygium recurrence after surgery have been studied by AS-OCT.

Therefore, the main purpose of this study is to identify predictive factors of recurrence after pterygium surgery with conjunctival autograft and tissue glue by imaging the pterygium preoperatively and imaging the conjunctival graft for one year postoperatively. Our hypothesis is that some tomographic characteristics of the pterygium or of the graft, not measurable with SLE, may be associated with an increased risk of recurrence.

The main conclusion of this study is that by imaging the graft with AS-OCT preoperatively and seven days after surgery (measuring the graft thickness), the risk of future recurrence can be predicted. This is significant because it could help to establish a standardized follow-up protocol in patients who undergo pterygium surgery in centres with AS-OCT; this protocol would aim to identify high-risk patients in order to individualise their follow-up and treatment.

## 2. Materials and Methods

### 2.1. Study Design

In this observational, prospective, and analytical study, 50 patients at an ophthalmology tertiary centre were considered for inclusion.

The study was conducted in accordance with the Declaration of Helsinki and approved by the Ethics Committee of Drug Research Ethics Committee of the CEI—Hospital Universitario y Politécnico La Fe (protocol code, 2020-294-1; date of approval, 10 August 2020) and the CEI—Fundación de Oftalmología Médica de la Comunidad Valencina (FOM) (protocol code, PI 107; date of approval, 30 September 2021).

### 2.2. Inclusion Criteria

Patients with primary pterygium were included. Patients with recurrent pterygium, presence of other relevant corneal or conjunctival pathology not related to pterygium that may influence the corneal topography, with other ocular treatments (excluding artificial tears) lasting more than 1 month, previous ocular surgery, recurrent pterygium, or younger than 18 or older than 70 years of age were excluded.

### 2.3. Treatment Protocol

Standard pterygium excision and conjunctival autograft with tissue glue (Tissucol^®^, Baxter, Portugal) under local anaesthesia was performed by the same surgeon on all patients, as it is the technique that has shown the lowest recurrence rate (2–20%) [11,12,13,14,15,16,17,18,19,20], and current evidence supports pterygium excision with conjunctival autograft fixation using fibrin glue followed by patching until the first postoperative visit [21].

The surgical technique used is detailed below. First, the limits of the lesion and the superior conjunctival graft of the same eye were marked, and both areas were infiltrated with subconjunctival anaesthesia (0.2 mL of lidocaine 2% with adrenaline 1/200,000). Then, the fibrovascular axis of the pterygium and corneal debris were freed by crescent knife keratectomy of the superficial cornea to achieve a dissection plane without residual steps. This was followed by resection of the pterygium and dissection of the perilesional tenon’s capsule. In this way we obtained a recipient area with regular edges free of tenon’s capsule, and we obtained an exposed, parallelogram-shaped scleral bed of approximately 8 × 4 + −2 mm (depending on the dimensions of the pterygium). The next step was to obtain, with Westcott scissors, a tenon-free conjunctival graft with dimensions 1 mm larger than the scleral bed on all sides to ensure its total coverage without tension and, then, to position this graft preserving the juxtalimbar-limbus orientation, with its long sides parallel to the limbus, in the same parallelogram shape as the bed. The manoeuvre consisted of releasing the perpendicular sides to the limbus and the superior parallel side to flip the graft over the cornea with the basal side upwards. Then, limbus side was released, and the graft was slipped over the cornea, preserving the juxtalimbar-limbus orientation. Once the flipped graft was placed over the cornea in the desired orientation, the two components of the tissue glue were sequentially applied so that the basal aspect of the graft finally faced the scleral bed.

In our study, no adjuvant therapies were used. At the end of surgery, ocular occlusion was performed for 24 h as recommended [21].

Postoperative use of topical steroids described in the literature is highly variable because there is no consensus regarding the optimal dose, frequency, and duration of treatment or its correlation with recurrence [21]; thus, after uncovering, the treatment recommended by the Spanish Society of Ophthalmology [11] was started:

1. Compressive occlusion for 24–48 h with antibiotic and corticosteroid ointment.
2. Anti-inflammatory eye drops: Dexamethasone 0.1% for 5 weeks: every 3 h × 7 days; every 6 h × 7 days; every 8 h × 7 days; every 12 h × 7 days; every 24 h × 7 days; and discontinue.
3. Ofloxacin 0.3% eye drops for one week every 8 h.
4. Preservative-free artificial tears every 2/3 h during the first month and then every 4 h for 3 months.

### 2.4. Clinical Assessment Protocol

The data used in this study were obtained during six control visits (one preoperative and five postoperative) using a specific standardised protocol that included the following steps:

1. First, 4 variables were obtained from the SLE assessment carried out in routine clinical practice (preoperatively, classifying the pterygium as atrophic, intermediate, or fleshy, and postoperatively, identifying graft alterations, the presence or absence of recurrence, and pattern of recurrence, as detailed below).
2. Then, 10 anatomic variables were obtained from the AS-OCT protocol “PTERIGIUM” designed specifically for this study, which includes the global scan and 2D analysis (for manual measurement of 10 anatomical variables of the pterygium or graft, as detailed below) and the topographic axial power map (to obtain the keratometric, posterior, and real map, for future analysis).

AS-OCT measurements were obtained as follows:

1. Drawing a line perpendicular to the ocular surface at the level of the scleral spur.
2. The intersection of this line with the ocular surface is the reference point from which measurements, towards the cornea or towards the bulbar conjunctiva or graft, were obtained.

At the preoperative visit (T0), the following parameters were measured by AS-OCT: limbus thickness (LimbusT), central pterygium thickness (CentreT), head pterygium thickness (HeadT), horizontal corneal invasion (Horizontal Corneal Inv), epithelial thickness at 1 mm (EpitT1mm), stromal thickness at 1 mm (stromT1mm), total thickness at 1 mm (TotalT1mm), total thickness at 2 mm (TotalT2mm), total thickness at 3 mm (TotalT3mm), pterygium type, and presence/absence of satellite masses. Pterygium type was classified as atrophic (vascularization below the body of the pterygium), intermediate (intermediate characteristics between the other two), or fleshy (vascularization in the body of the pterygium) with SLE and as nodular (when the subepithelial mass causes a convex change in the curvature of the ocular surface at the limbus) or flat/continuous (the shape of the ocular surface contour is not altered, and there is a hypodense space with the appearance of a “void” in the sclerocorneal angle) with AS-OCT.

After surgery, patients were re-evaluated at 10 +/− 5 days (T1), 1 month +/− 5 days (T2), 3 months +/− 5 days (T3), 6 months +/− 5 days (T4), and 1 year +/− 5 days (T5). At all times, the following were measured: epithelial graft thickness at 1 mm (EpitT1mm), stromal thickness at 1 mm (StromT1mm), total thickness at 1 mm (TotalT1mm), total thickness at 2 mm (TotalT2mm), total thickness at 3 mm (TotalT3mm), and presence/absence of remnant tissue with AS-OCT. Graft alterations, presence/absence of recurrence (defined as any tissue growth over the cornea, regardless of its extent or need for reintervention) and recurrence pattern (1: atrophic, 2: intermediate or 3: fleshy) were assessed with SLE.

All variables were measured by the same researcher and in the same time range (between 9 am and 2 pm).

### 2.5. Statistical Analysis

Statistical analysis was performed using SPSS software, version 21.0 (SPPS Inc., Chicago, IL, USA) for descriptive variables and R-project (The R foundation©, version 3.0.2) for the rest of the analyses. The occurrence of recurrence is the primary outcome of the investigation. The significance level used in the analysis was 5% (α = 0.05).

## 3. Results

### 3.1. Descriptive Analysis

The study involved 25 men (50%) and 25 women (50%), with a mean age of 43 ± 9.5 years and a range of 27 to 61 years. In most cases, the pterygium was only nasal (98%), while 2% had pterygium in both nasal and temporal locations. Of the participants’ eyes, 52% had unilateral pterygium, 48% had bilateral pterygium, 60% had an intermediate pattern, 32% had a fleshy pattern, 8% had an atrophic pattern with SLE, 62% had a nodular pattern, and 38% had a flat pattern with AS-OCT. Satellite masses were identified in only two cases (4%). Regarding the description of the thickness in the limbus, centre, and head before the preoperative time, the median thickness at the limbus was 0.547 (IQR: 0.449–0.626), the median thickness at the centre was 0.520 (IQR: 0.421–0.649), and the median thickness at the head was 0.187 (0.148–0.235). The respective means were 0.571, 0.549, and 0.187 (mm). Eight patients presented recurrence (16% rate with 95%CI: 5.8–26.2%). Recurrences were recorded at the following times: at 1 month, there was one recurrence; at 3 months, there were six recurrences; and at 6 months, there was one recurrence. Since most of the recurrences appeared after 1 month, interest was focused on the predictive value of the variables at the preoperative time and at 1 week. Five (62.5%) of the recurrences were type 1 (atrophic) and three (37.5%) were type 3 (fleshy); 18% of the samples had a corneal remnant.

### 3.2. Complication Rates

Regarding postoperative alterations, intragraft haemorrhages (eight cases; 47.1%) and carbuncular granulomas (four cases; 23.5%) were the most frequent citations, while there was only one patient with graft necrosis.

### 3.3. Prognostic Factors of Recurrence

We considered the pterygium recurrence outcome (yes/no) and evaluated the association between recurrence rate and the different independent variables (one by one) in Table 1. Some relevant associations have been detected, all referring to conjunctival thickness (preoperatively and postoperatively).

#### 3.3.1. Preoperatively

- When the total TotalT2mm at T0 increases by 1 micron, the odds of recurrence (equivalent to the probability) is multiplied by 1.005; i.e., the risk increases by 0.5%. In other words, an increase of 10 microns increases the risk by 5%. This relationship is very close to statistical significance (*p* = 0.055).
- When the total TotalT3mm at T0 increases by 1 micron, the odds of recurrence are multiplied by 1.008; i.e., the risk increases by 0.8%. In other words, an increase of 10 microns increases the risk by 8%. This relationship is statistically significant (*p* = 0.029).

No other preoperative variables were associated with subsequent recurrence. It should be noted that no atrophic case presented recurrence. The rates with intermediate and fleshy typology were 18.5% and 15.8% which, although higher, are not sufficient to speak of significant dependence (*p* = 0.641, Chi^2^).

#### 3.3.2. Postoperatively

- When the epithelial EpitT1mm in T1 increases by 1 micron, the odds of recurrence are multiplied by 1.014; i.e., the risk increases by 1.4%. In other words, an increase of 10 microns increases the risk by 14%. This relationship is very close to statistical significance (*p* = 0.061).
- When the stromal stromT1mm in T1 increases by 1 micron, the odds of recurrence are multiplied by 1.008; i.e., the risk increases by 0.8%. In other words, an increase of 10 microns increases the risk by 8%. This relationship is statistically significant (*p* = 0.003).
- When the total TotalT3mm in T1 increases by 1 micron, the odds of recurrence (equivalent to the probability) are multiplied by 1.016; i.e., the risk increases by 1.6%. In other words, an increase of 10 microns increases the risk by 16%. This relationship is statistically significant (*p* = 0.006).

In view of the results and the expected correlation between thickness measurements of the same patient, a multiple model is proposed for the relevant parameters at T0 and another for those relevant at both T0 and T1.

For the model at T0, the potential parameters were total thickness at 2 mm and at 3 mm. The association between recurrence and relevant lesion parameters at this time (results of the binary logistic regression model for the probability of recurrence and the estimation of odds ratio (OR) adjusted with the stepwise method of variable entry in the model) is shown in Table 2. Total thickness at 3 mm is the only significant variable. Knowing the value of this parameter and knowing the thickness at 2 mm does not contribute anything new, and it does not improve the predictability of recurrence.

Regarding the model for parameters at T0 and T1, these are shown in Table 3. These include the two previously mentioned variables, epithelial and stromal thickness at 1 mm and total thickness at 3 mm at T1 and the association between recurrence and relevant lesion parameters at T0 and T1 (the results of the multiple binary logistic regression model for probability of recurrence and the estimation of the odds ratio (OR) adjusted with the stepwise method of variable input in the model). The pair of variables included in the model (StromT1mm and TotalT3mm) are the most relevant for the prediction of recurrence. It is reasonable that if there is any correlation between them, their simultaneous inclusion in the model makes neither of them reach significance, although the trend is strong.

**Figure 1:**
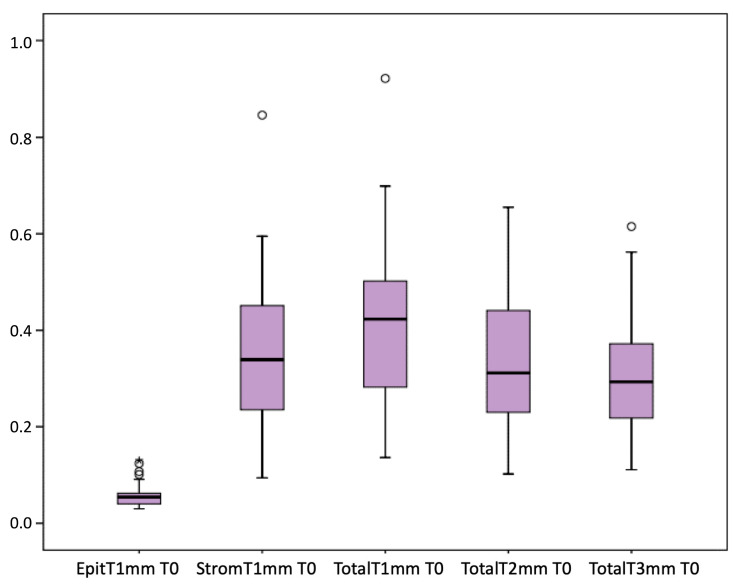
Full basal description of conjunctival thickness.

**Figure 2:**
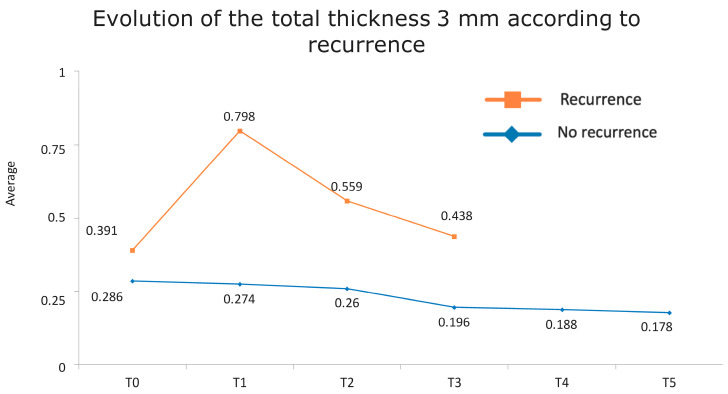
Evolution of the total thickness at 3 mm according to recurrence.

**Figure 3:**
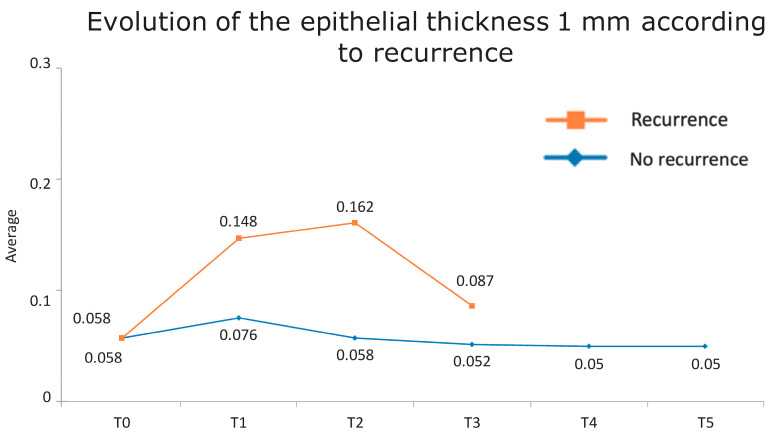
Evolution of the epithelial thickness at 1 mm according to recurrence.

**Figure 4:**
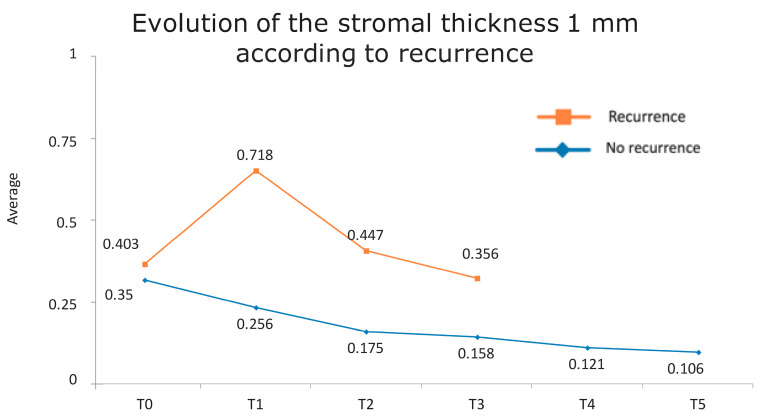
Evolution of the stromal thickness at 1 mm according to recurrence.

Figure 1 presents the full basal description of conjunctival thickness. Evolution of the significant variables (TotalT3mm and StromT1mm) and almost-significant variables (EpitT1mmover time) according to recurrence is represented in Figure 2, Figure 3, and Figure 4, respectively.

### 3.4. Predictive Ability of the Models

**Figure 5:**
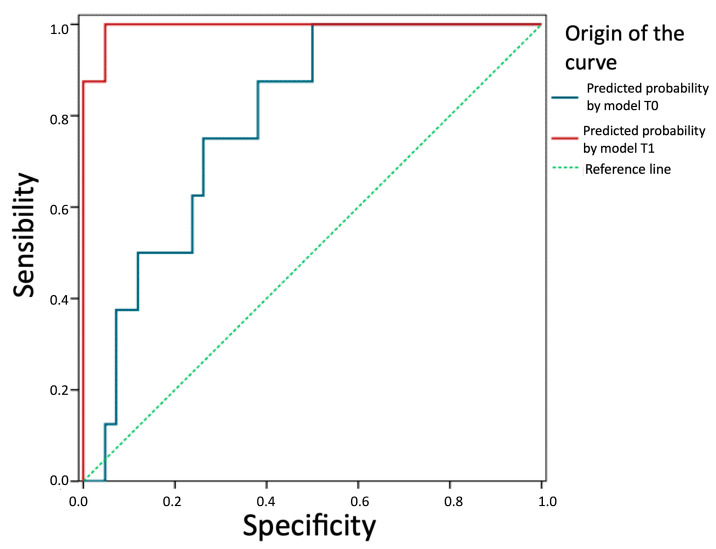
Comparison of the predictive ability of the two models. Predictive probability by model T1 (StromT1mm and TotalT3mm 1-week post-intervention), predictive probability by model T0 (TotalT3mm preoperatively), and reference line (broken line) are represented from left to right, respectively, showing that both models are good prognostic factors, especially the prediction based on the model with parameters from 1-week post-intervention.

We evaluate and compare the predictive ability of the two models in Figure 5. The prediction of future recurrence based on the logistic equation of the T0 model (TotalT3mm) presents an AUC = 0.79 (95%CI: 0.65–0.93), which is significantly above that expected by chance (*p* = 0.010). Therefore, this thickness can be considered a good prognostic factor. The prediction based on the model with parameters from 1-week post-intervention is even better (AUC = 0.99; 95%CI: 0.98–1.00; *p* < 0.001). In fact, a z-test for AUC comparison of both models concludes that the one based on post-intervention parameters is more effective (z = 2.87; *p* = 0.006).

**Figure 6:**
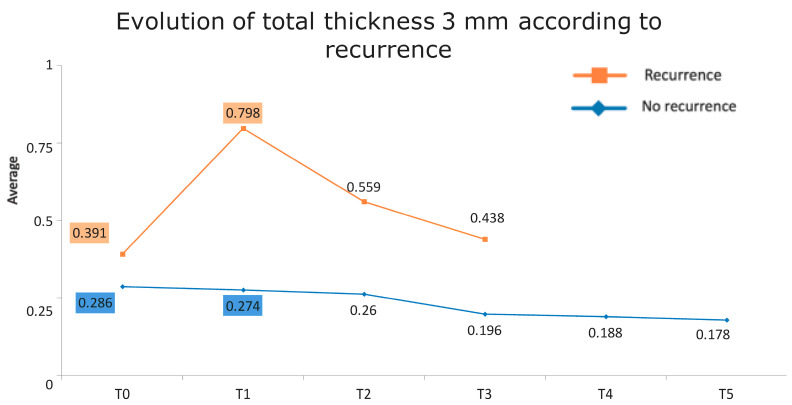
Evolution of the total thickness at 3 mm according to recurrence. The vertical axis represents the average thickness in mm, and the horizontal axis represents time. The mean values corresponding to the most discriminating parameters that have been integrated into the models have been highlighted (TotalT3mm preoperatively and 1-week post-intervention). Note the large difference in the averages of patients who did (square) and did not (rhombus) develop recurrences.

**Figure 7:**
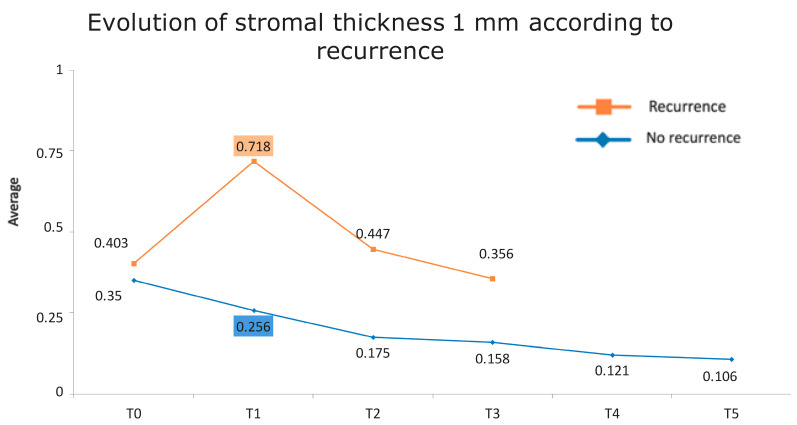
Evolution of stromal thickness at 1 mm according to recurrence. The vertical axis represents the average thickness in mm, and the horizontal axis represents time. The mean values corresponding to the most discriminating parameters that have been integrated into the models have been highlighted (StromT1mm one week after surgery). Note the large difference in the averages of patients who did and did not develop recurrences.

All these results are fully consistent with what was seen in the descriptive analysis, as is highlighted in Figure 6 and Figure 7.

## 4. Discussion

AS-OCT enables measurement of the actual size, thickness, and structure of pterygia [22] and its differential diagnosis with other ocular surface pathologies such as ocular surface squamous neoplasia [23] and corneo-conjunctival intraepithelial neoplasia [24].

In the study of pterygium through AS-OCT images, it has been observed that some features could be prognostic. For example, different patterns of pterygium (flat or nodular) measured by AS-OCT are associated with different factors (the flat pattern is associated with greater corneal astigmatism and stromal scarring) [10]. Preoperative satellite masses of pterygium tissue below the epithelium and the clinically visible pterygium margins, that might be related to recurrence, have been described by AS-OCT [25]. It has also been observed how AS-OCT performed immediately after excision can show the existence of remnants on both the corneal and conjunctival sides, which could elucidate the importance of resecting and smoothing the corneal and conjunctival sides in pterygium surgery [25]. AS-OCT is useful after pterygium surgery to understand the mechanism of tissue repair or recurrence, thereby predicting the final outcome of surgery [10]. The evolution of postoperative conjunctival graft thickness studied by AS-OCT shows a significant thickening of the graft the first week after surgery which then decreases until 3 months and shows the presence of a greater conjunctival graft thickness in recurrent pterygium surgery than in primary pterygium surgery the first month after surgery [6,26]. AS-OCT has also been used to measure graft thickness in order to compare different surgical techniques [27]. However, until now, the influence of graft thickness or other characteristics on recurrence had not been studied.

Regarding our study design, the same surgical technique was performed by the same surgeon, and the same postoperative treatment was prescribed in all patients in the study. In addition, all AS-OCT measurements were obtained by the same researcher and in the same time range. These facts confer reliability to the results. The technique chosen was conjunctival autograft surgery, as it has shown the lowest recurrence rates [1] and best cosmetic results [6], and graft fixation by tissue glue, as it reduces intraoperative time and it is the one that produces less irritation and discomfort in patients [28,29].

With regard to the results of the study, several facts should be highlighted. On the one hand, our recurrence rate is 16%, which is to be expected, since the recurrence rate described in the literature using the same technique is between 2 and 20% [11,12,13,14,15,16,17,18,19,20]. It is important to mention that recurrence can be measured by clinical criteria or by need for reintervention. In our work, we considered recurrence according to clinical criteria, considering any fibrovascular growth on the cornea as a recurrence, and no recurrence was clinically significant enough to require reintervention during the first year of follow-up. These facts could explain why our recurrence rate is 16% and not lower. We consider it advantageous to use the clinical criteria for recurrence, as it allows us to be more sensitive when detecting incipient recurrences in order to use nonsurgical medical treatments (such as intralesional subconjunctival injections of 5-FU or anti-VEGF in subconjunctival or topical injection) [30] that act as modifying factors in the evolution of the disease and prevent future surgical reintervention. In addition, most of the recurrences were 3 months after the intervention, which is consistent with the published literature [6,7,8,10]. On the other hand, 18% of the samples had corneal remnants, which was not significantly associated with recurrence, contrary to what previous publications suggest [25]. Finally, our work provides several facts not previously studied or described in the literature, among which the following can be highlighted:

- Total thickness at 3 mm preoperatively appears significantly increased in patients who developed recurrence.
- Epithelial and stromal thickness at 1 mm and total thickness at 3 mm one week after surgery are useful for predicting recurrence.

As a weakness of our study, it is worth mentioning that, preoperatively, the thickness and horizontal invasion length of the pterygium over the cornea were measured, but the radial length of the pterygium over the limbus was not measured, which could also influence the recurrence rate. Also, the stromal thickness of the graft could be influenced by the surgical technique used to obtain a graft with a greater or lesser amount of stroma, and both epithelial and stromal thicknesses could be influenced by postoperative inflammation. This postoperative inflammation could be of preoperative origin (as the overexpression of several proteins has been observed on the ocular surface of eyes with pterygium, such as defensins, phospholipase D, and positive regulation of growth factors, including basic fibroblastic growth factor bFGF or vascular endothelial growth factor VEGF) [31,32,33] or intraoperative origin (due to a more aggressive surgical technique). Therefore, it would be interesting to measure preoperative and postoperative tear film inflammatory markers in future studies to determine the origin of this inflammation.

Another point worth mentioning is that although surgical adjuvants and postoperative use of artificial tears and topical cyclosporine 0.05% may further reduce recurrence [21], only artificial tears were included in our study; therefore, it would be interesting to introduce cyclosporine and compare the recurrence rate with a control group in future studies. Future studies should also examine whether there is an association between the anatomical characteristics of the pterygium and the topographical variables included in the AS-OCT protocol of this study.

## 5. Conclusions

The main finding of this study is that between the factors that showed statistically significant association with recurrence, those that showed the best predictive capacity were the stromal thickness of the graft at 1 mm and the total thickness of the graft at 3 mm at 7 days after surgery. Therefore, by imaging the graft with AS-OCT at 7 days after surgery, the patient’s risk of recurrence can be predicted. This is clinically relevant because it allows the establishment of an individualised follow-up protocol for patients who have undergone pterygium surgery according to their risk of recurrence using a single test without discomfort [34] for the patient. This test is becoming increasingly widespread and available in healthcare centres.

## Figures and Tables

**Table 1:** Association between recurrence and clinical/anatomical profile of the patient and the lesion at T0 and T1: results of simple binary logistic regression model for probability of recurrence. Estimation of unadjusted odds ratio (OR). Chi^2^ test and Fisher’s exact test when OR not estimable.

	Category	OR	IC 95%	*p*-Value
**Gender**	Male	1		0.376
	Female	2.02	0.43–9.55	
**Age**		0.98	0.83–1.15	0.806
**Eye**	Left	1		0.687
	Right	0.73	0.15–3.44	
**Bilaterality**	No	1		0.519
	Yes	0.6	0.13–2.84	
**Atrophic/intermediate/fleshy**	Atrophic	---		0.641 (Chi^2^)
	Intermediate	1		
	Fleshy	0.83	0.17–3.96	0.810
**Nasal/temporal**	---			
	---		---	1.000 (Fis)
**Nodular/flat**	Nodular	1		
	Flat	2.19	0.4 -19.9	0.488
**LimbusT T0**		1.001	0.997–1.005	0.713
**CentreT T0**		0.999	0.995–1.004	0.729
**HeadT T0**		0.990	0.977–1.003	0.137
**Horizontal corneal invasion**		1.000	0.999–1.001	0698
**EpiT1mm T0**		1.001	0.969–1.034	0.967
**StromT1mm T0**		1.002	0.997–1.007	0.380
**TotalT1mm T0**		1.002	0.997–1.006	0.410
**TotalT2mm T0**		1.005	1.000–1.011	0.055
**TotalT3mm T0**		1.008	1.001–1.014	0.029 *
**Satellite mass T0**	No	---		
	Yes	---	---	0.529 (Fis)
**EpitT1mm T1**		1.014	0.999–1.029	0.061
**StromT1mm T1**		1.008	1.003–1.013	0.003 **
**TotalT1mm T1**		1.045	0.989–1.103	0.118
**TotalT2mm T1**		1.037	0.987–1.090	0.149
**TotalT3mm T1**		1.016	1.005–1.028	0.006 **
**Remnant**	No	1		
	Yes	0.71	0.08–6.76	0.769
**Graft alterations**	No	1		
	Yes	0.86	0.09–8.27	0.894

* *p* < 0.05; ** *p* < 0.01.

**Table 2:** Association between recurrence and relevant lesion parameters at T0: results of multiple binary logistic regression model for probability of recurrence. Estimation of odds ratio (OR) adjusted with stepwise method of variable entry in the model.

	OR	IC 95%	*p*-Value
**TotalT3mm T0**	1.008	1.001–1.014	0.029 *

* *p* < 0.05.

**Table 3:** Association between recurrence and relevant lesion parameters at T0 and T1 (results of multiple binary logistic regression model for probability of recurrence and estimation of odds ratio (OR) adjusted with stepwise method of variable input in the model).

	OR	IC 95%	*p*-Value
**StromT1mm T1**	1.015	0.997–1.032	0.097
**TotalT3mm T1**	1.021	0.998–1.046	0.079

## Data Availability

There were no new data created.
